# Phase I study of S-1, docetaxel and cisplatin combination chemotherapy in patients with unresectable metastatic gastric cancer

**DOI:** 10.1038/sj.bjc.6603957

**Published:** 2007-09-11

**Authors:** T Takayama, Y Sato, T Sagawa, T Okamoto, H Nagashima, Y Takahashi, H Ohnuma, G Kuroiwa, K Miyanishi, R Takimoto, T Matsunaga, J Kato, K Yamaguchi, K Hirata, Y Niitsu

**Affiliations:** 1 Fourth Department of Internal Medicine, Sapporo Medical University, School of Medicine, Sapporo, Japan; 2 Department of Gastroenterology, Hokkaido Cancer Center, Sapporo, Japan; 3 Department of Internal Medicine, Higashi Sapporo Hospital, Sapporo, Japan; 4 First Department of Surgery, Sapporo Medical University, School of Medicine, Sapporo, Japan

**Keywords:** gastric cancer, S-1, docetaxel, cisplatin, downstaging, VEGF

## Abstract

The aim of this dose escalation study was to determine the maximum-tolerated dose (MTD), dose-limiting toxicities (DLTs) and preliminary efficacy of docetaxel, S-1 and cisplatin combination chemotherapy in patients with unresectable metastatic gastric cancer. Seventeen patients received oral S-1 (40 mg m^−2^ bid) on days 1–14, intravenous cisplatin (60 mg m^−2^) and docetaxel (60, 70 or 80 mg m^−2^ depending on DLT) on day 8 every 3 weeks. The MTD of this combination was presumed to be docetaxel 70 mg m^−2^. At this dose level, 40% of the patients (two of five) developed grade 4 neutropenia and 20% (one of five) exhibited grade 3 nausea during the first course. Therefore, the recommended dose of docetaxel was defined as 60 mg m^−2^. The DLT was neutropenia. The response rate (RR) was 88.2% (15 of 17), consisting of one complete response and 14 partial responses. There were two stable diseases but no progressive disease. Of these 15 responders, four (23.5%) with high VEGF expression showed rapid tumour regression and achieved downstaging, leading to subsequent curative gastrectomy. Three of these have been disease free for about 3 years, suggesting a complete cure. In conclusion, this regimen was tolerable and showed a quite high RR, with an appreciable downstaging rate in metastatic gastric cancer.

Although the incidence of gastric cancer is declining in Western countries, it is still the fourth most common malignancy worldwide (Konturek *et al*, 2006). Except in the countries such as Japan and Korea, where early detection programs are in place, the disease is often diagnosed after it becomes metastatic or presents at a locally advanced stage, and chemotherapy is the only potential treatment for such advanced gastric cancer.

Over the past several decades, 5-FU-based regimens, either in combination with other drugs or in monotherapy, have been accepted as standard chemotherapy for unresectable gastric cancer, since they have shown a significant survival benefit compared with the best supportive care (Murad *et al*, 1993; Pyrhonen *et al*, 1995; Glimelius *et al*, 1997). However, overall response rates (ORRs) and median survival times (MSTs) of these regimens, even in combination therapy, have been only 7–51% and 6–12 months, respectively (Dickson and Cunningham, 2004; Shah and Schwartz, 2004; Ohtsu *et al*, 2006).

The recent introduction of a new oral drug, S-1 (Shirasaka *et al*, 1996), which was developed on the basis of biochemical modulation to inhibit dihydropyrimidine dehydrogenase and orotate phosphoribosyltransferase to therapeutic modality for gastric cancer, has enabled us to increase ORR to 46–74% and MST to 10.9–14.8 months by its combination with cisplatin (CDDP), docetaxel or CTP-11, with less toxicity than 5-FU (Koizumi *et al*, 2003; Ajani *et al*, 2006; Inokuchi *et al*, 2006; Yamaguchi *et al*, 2006).

Another relatively new drug, docetaxel, has also proven to be quite active against gastric cancer, with ORR ranging from 17 to 24% as a single agent (Sulkes *et al*, 1994; Einzig *et al*, 1996). In combination with CDDP, 5-FU or CDDP plus 5-FU or S-1, docetaxel has shown a higher ORR of 37 to 56% and MST of 9.0 to 14.3 months (Roth *et al*, 2000; Thuss-Patience *et al*, 2005; Van Cutsem *et al*, 2006; Yamaguchi *et al*, 2006).

In the present study, we conducted a phase I study of triplet combination with S-1, docetaxel and CDDP for the treatment of unresectable metastatic gastric cancer. In this study, we determined the maximum-tolerated dose (MTD), dose-limiting toxicity (DLT) and recommended dose (RD), and also examined preliminary therapeutic activity of this combination.

## MATERIALS AND METHODS

### Patient selection

Patients were entered into the study if they fulfilled the following eligibility criteria: (1) histologic confirmation of gastric cancer; (2) unresectable distant metastatic disease (M1 stage according to the Japanese Classification of Gastric Carcinoma; Nishi *et al*, 1999); (3) measurable lesion(s) or evaluable disease; (4) age⩽75 years; (5)performance status (PS)⩽2 on the Eastern Cooperative Oncology Group (ECOG) scale; (6) no prior chemotherapy; (7) adequate bone marrow function (WBC count⩾4000 ml^−1^ and platelet count⩾100 000 ml^−1^); (8) adequate liver function (serum bilirubin level⩽1.5 mg dl^−1^ and serum transaminase levels ⩽three times the upper limit of normal); (9) adequate renal function (serum creatinine level⩽1.5 mg dl^−1^, blood urea nitrogen level⩽25 mg dl^−1^ and creatinine clearance⩾50 ml min^−1^.); (10) no other severe medical conditions; (11) not pregnant or lactating and (12) provision of written informed consent in accordance with government guidelines (Good Clinical Practice, by the Ministry of Health and Welfare of Japan) and guidelines of each institution or hospital. This study was approved by the ethics committees in each institution or hospital.

### Treatment and dose escalation schedule

S-1 was given orally at a dose of 40 mg m^−2^ twice daily on days 1–14, followed by a 7-day recovery period. CDDP was administered by intravenous infusion for 120 min at a dose of 60 mg m^−2^ on day 8. The starting dose of docetaxel was 60 mg m^−2^ (level 1), which was planned to be increased in 10 mg m^−2^ increments to 80 mg m^−2^. The starting dose of docetaxel corresponded to 80% of the recommended dose of the TCF regimen for gastric cancer reported by Ajani *et al* (2005a). No intrapatient dose escalation was allowed. At least three patients were treated at each dose level. This treatment course was repeated every 3 weeks, with an allowance for a delay in treatment if toxicity was observed. To avoid CDDP-induced renal damage, patients were hydrated on days 7–9 with 2000 ml 5% dextrose in 0.9% sodium chloride. Prophylactic administration of antiemetic medication (5-HT_3_ antagonist plus corticosteroid) at a standard dose was routinely used to prevent nausea and vomiting when CDDP was administered. G-CFS was administered only when grade 4 neutropenia lasting for 3 days, or grade 3 or 4 neutropenia with fever (DLTs as described bellow) had been observed. The treatment was repeated unless disease progression was observed. When patients underwent downstaging and were deemed able to tolerate a curative surgical operation, subsequent gastrectomy with lymph node dissection was performed.

### Evaluation of the disease

Before a patient could enter the study, the extent of the disease was determined by physical examination, chest X-ray, gastrointestinal X-ray, endoscopic examination of the upper gastrointestinal tract, abdominal ultrasonography, computed tomographic scan of the abdomen, barium enema and bone scintiscan. Peritoneal metastasis was cytologically confirmed by abdominal ascites puncture or culdocentesis. Complete blood cell counts, liver function tests, renal function tests and urinalysis were assessed at least once per week during treatment. If grade 4 neutropenia was noted, the neutrophil count was repeated 2 days later to determine whether the grade 4 neutropenia had lasted for 3 days or longer. Computed tomographic scanning and imaging of measurable disease were carried out in every cycle or once in every two cycles. Tumour response of metastatic lesions was evaluated according to WHO criteria (World Health Organization, 1979). A complete response (CR) was defined as the disappearance of all evidence of cancer for ⩾4 weeks. A partial response (PR) was defined as ⩾50% reduction in the sum of the products of the perpendicular diameters of all lesions for 4 weeks, without any evidence of new lesions or progression on any lesions. No change (disease stabilization) was defined as less than a 50% reduction or less than a 25% increase in the sum of the products of the perpendicular diameters of all lesions, without any evidence of new lesions. Progressive disease (PD) was defined as a more than 25% increase in ⩾1 lesion or the appearance of new lesions. Tumour responses of the primary site were evaluated by the roentgenographic and endoscopic evaluation criteria proposed by the Japanese Research Society for Gastric Cancer (Nishi *et al*, 1999). Time to progression (TTP) was defined as the number of days from the start of treatment to the onset of any progression or until death. Overall survival (OS) was defined as the number of days from the start of treatment to death. Downstaging was defined as the disappearance of all lesions of distant metastases (M0 stage) for ⩾4 weeks. All responses were reviewed by two external review panels.

Toxicities were graded according to the National Cancer Institute Common Toxicity Criteria (NCI-CTC) Version 2.0. The DLT was defined as one of the following: (1) grade 4 neutropenia lasting more than 3 days, or grade 3 or 4 neutropenia with fever; (2) grade 4 thrombocytopenia; (3) grade 3 non-haematological toxicity and (4) treatment delay of greater than 2 weeks as a result of toxicity. Dose-limiting toxicity was assessed during the first course of treatment. The MTD was defined as the dose at which 33% or more patients experienced DLTs during the first course. If the patients who developed DLT showed response (⩾50% reduction in the sum of the products of the perpendicular diameters of all lesions), the subsequent cycle was started at the next lower level after complete recovery from the toxic effect of the previous cycle.

### Immunohistochemistry for VEGF

Paraffin-embedded tissue sections of gastric cancer tissue were deparaffinized in xylene and treated for 20 min with 0.6% H_2_O_2_ to block endogenous peroxidase activity. They were incubated overnight at 4°C in a 1 : 100 dilution of rabbit polyclonal antibody against VEGF (clone A-20, Santa Cruz Biotechnology, Santa Cruz, CA, USA). Binding of the primary antibody was detected by peroxidase staining with an avidin–biotin complex system (Dako, Carpinteria, CA, USA).

We classified VEGF staining as negative, weak positive or strong positive according to the percentage of positive cells and staining intensity by the method of Gong *et al* (2005), with a minor modification. In brief, scores for percentage of positive cells were assigned as follows:⩽10% of cells positive, 0; 11–25% of cells positive, 1; 26–50% of cells positive, 2; 51–75% of cells positive, 3 and >75% of cells positive, 4. Scores for staining intensity were assigned as follows: no staining, 0; light brown, 1; brown, 2 and dark brown, 3. Overall scores were obtained by multiplying the percentage score by the intensity score. Overall scores ⩽5 were defined as negative, overall scores >5 but ⩽15 were defined as weak positive and overall scores >15 were defined as strong positive. Two independent pathologists examined five random fields (300 *μ*m^2^) of each sample and scored each sample without knowledge of patient outcome (double blind). An average value of the two scores was presented in the present study.

## RESULTS

### Patient characteristics

Between December 2002 and November 2004, 17 patients were enrolled in this study. No patients had received prior chemotherapy or undergone surgical gastrectomy. All patients were assessable for toxicity and response. Their characteristics are summarized in Table 1. There were 12 men and five women, with a median age of 61 years (range 54–75 years). Five patients were PS 0, 11 PS 1 and one PS 2. Histologically, the cancer was of intestinal type in six patients and diffuse type in 11. Lymph node metastases were seen in all 17 patients, and 14 of 17 patients had distant lymph node metastases consisting of seven with para-aortic lymph node, five with Virchow lymph node and two with mediastinal lymph node metastases. Of these 14 patients, eight had additional distant metastases to liver (2), peritoneum (3) and bone (3). All three patients with local lymph node metastases had distant metastases to liver (2), and lung and peritoneum (1). In other words, distant metastases other than lymph node were found in the liver of four patients, lung of one patient, bone of three patients and peritoneal cavity of four patients (one had both lung and peritoneal metastases).

### Toxicities

The first cohort of three patients was entered on level 1, and no DLTs were observed. The next cohort of three patients received dose level 2, and one patient experienced grade 3 neutropenia and grade 2 anorexia, although none of them developed DLT at this point. Therefore, the next three patients were entered on dosage level 3. At this level, two of the three patients (66.7%) experienced grade 4 neutropenia and leukocytopenia, which lasted for more than 3 days, and grade 3 nausea. Therefore, in order to confirm the safety of level 2, two additional patients were entered on this level. However, both exhibited DLT; one exhibited grade 4 neutropenia lasting for 3 days, as well as grade 3 anorexia. The other developed grade 4 neutropenia lasting for more than 5 days and required more than 14 days (20 days) to start the second course. In total, multiple DLTs of grade 4 neutropenia with grade 3 nausea or treatment delay occurred in two of the five patients (40%) at level 2.

In order to confirm the safety of level 1, an additional six patients were enrolled in the level 1 group. Of the nine patients treated at level 1, no grade 4 neutropenia was observed in the first course, although grade 3 neutropenia and leukocytopenia were observed in three of the nine patients (33%), grade 2 anaemia in one of the nine (11%) and grade 2 nausea in three of the nine (33%) (Table 2). Based on these results during the first course of treatment, we concluded that the MTD and RD with this regimen were level 2 and level 1, respectively, and that the DLT was neutropenia.

The total number of treatment courses was 80 (51 courses at level 1, 22 courses at level 2, and 7 courses at level 3). The toxicities observed during all courses are summarized in Table 3. Neutropenia was the most commonly observed haematological toxicity. No grade 3/4 anaemia or thrombocytopenia occurred in any dose levels. At levels 2 and 3, grade 4 neutropenia was frequently observed. At level 1, two of the nine patients (22.2%) developed grade 4 neutropenia after four or five courses, respectively. In terms of non-haematological toxicities, grade 3 nausea was observed in one of the five (20%) at level 2 and two of the three (66.7%) at level 3. At level 1, no grade 3 nausea or other toxicities were observed. The nadir of leukocyte and neutrophil counts occurred around day 17. Neither treatment-related death nor delayed severe toxicities was observed.

### Response

All 17 patients had measurable metastatic lesions. The response rates (RRs) in levels 1, 2 and 3 were 88.9 (PR 8/9), 80 (CR 1/5, PR 3/5) and 100% (PR 3/3), respectively (Table 4). The ORR was 88.2% (1 CR and 14 PR in 17 patients; 95% confidence interval, 63.6–98.5%). The RRs of the primary tumours, lymph node metastasis and liver metastasis were 82.4 (14/17), 88.2 (15/17) and 100% (4/4), respectively. Ascites disappeared in all four patients. The RRs for the intestinal type and the diffuse type were 100 (6 of 6) and 81.8% (9 of 11), respectively.

The most striking finding of this study is that four of the 17 patients (23.5%, 1 CR and 3 PR) achieved downstaging and underwent subsequent gastrectomy. Downstaging was seen at all dose levels. Of these four cases, three showed histologically intestinal type and one diffuse type. The CR patient had intestinal type of adenocarcinoma, which directly invaded into the liver, as revealed by a CT scan and ultrasonography, and had multiple distant (para-aortic) lymph node metastasis before treatment. After four courses of treatment at the level 2 dose, the tumour in the stomach completely disappeared, as determined by a gastrofiberscope examination; lymph node swelling as well disappeared. After five courses, he underwent total gastrectomy with lymph node dissection (D2) and partial hepatectomy and was proven to be histologically CR at all sites. Two of the three downstaged patients had multiple distant lymph node metastases, and the remaining patient had multiple liver metastases before treatment. In these four cases, the median time to response was 42 days, which was much less than that (65 days) in other PR cases. The metastatic lesions in all four patients completely disappeared after 2–5 treatment courses. The patients subsequently underwent surgical gastrectomy with lymph node dissection (D2). Three of those four patients (1 CR and 2 PR) have been disease free for about 3 years, suggesting a possible complete cure, and one patient showed a recurrence of liver metastasis 232 days after surgical gastrectomy, followed by second-line chemotherapy with CPT-11/CDDP (Shirao *et al*, 1997).

As a second-line therapy, seven received CPT-11/CDDP, three received S-1 monotherapy (Sakata *et al*, 1998), two paclitaxel monotherapy (Yamada *et al*, 2001) and one MTX/5-FU therapy (Murakami *et al*, 1987). The three-year survival rates were 23.5% (4/17) in all cases and 22.2% (2/9) in level 1. The median TTP was 199 days (range 104–1130 days) in all cases and 226 days (range 104–1130 days) in the level 1 group. The median OSs of these groups were 367 and 389 days, respectively. The median follow-up time for survival analysis was 1120 days.

### Expression of VEGF in gastric cancer tissues

**Figure 1:**
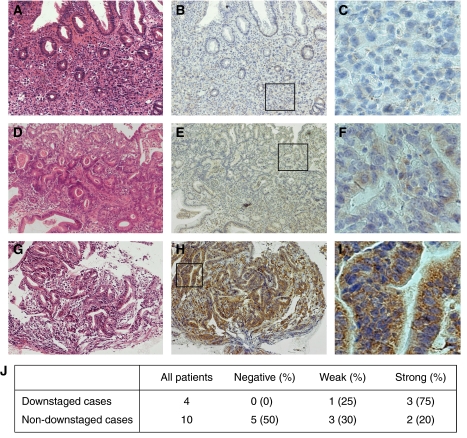
VEGF protein expression in gastric cancer tissue. Representative microphotographs of negative (**A**–**C**), weak (**D**–**F**) and high expression (**G**–**I**) of VEGF. Immunohistochemical staining for VEGF was performed using a specific antibody against VEGF. (**A, D, G**) H&E staining. (**B, E, H**) Immunostaining for VEGF. (**C, F, I**) Representative areas from (**B**) (**E**) and (**H**), respectively. The downstaged cases showed a significantly higher expression of VEGF than non-downstaged cases by Mann–Whitney *U*-test (*P*=0.043) (**J**).

Since docetaxel and S-1 have been suggested to show some antiangiogenic activity (Hironaka *et al*, 2002; Hotchkiss *et al*, 2002) and a rather rapid disappearance of lesions was observed in four downstaged patients, we examined the possible correlation between downstaging (marked therapeutic effect) and VEGF expression in the tumour tissues by immunohistochemical staining. In Figure 1, three representative staining patterns of negative (panels A–C), weak positive (panels D–F) and strong positive (panels G–I) are shown. The positive immunohistochemical staining for VEGF was observed in 4/4 of the downstaged cases, whereas it was only in 5/10 of the non-downstaged cases. When the staining grades (negative, weak positive, strong positive) were compared between these two groups, the downstaged cases showed a significantly stronger expression than non-downstaged cases (*P*=0.043 by Mann–Whitney's *U* test) (panel J).

## DISCUSSION

In this study, we conducted a phase I study of S-1, docetaxel and CDDP combination for 17 unresectable metastatic gastric cancer patients and defined RD as S-1 40 mg m^−2^ (twice daily, days 1–14), docetaxel 60 mg m^−2^ (day 8) and CDDP 60 mg m^−2^ (day 8). Non-haematologic toxicities including diarrhoea and nausea were relatively mild and none was greater than grade 3 at the dose of RD. The DLT was neutropenia. Grade 3/4 neutropenia occurred in 66.7% of patients in all treatment courses. However, it was generally manageable, and each treatment course could be performed as planned without a delay in most cases.

For combination chemotherapy regimens, which included S-1, either a 2-week administration with a 1-week interval protocol (Hyodo *et al*, 2003; Yoshida *et al*, 2006), or 3-week administration with a 1- to 2-week interval protocol have been reported (Koizumi *et al*, 2003; Ajani *et al*, 2005b). However, a recent post-marketing surveillance of S-1 disclosed that most toxicities increased during the third week of S-1 administration, often resulting in discontinuation of treatment (Nagashima *et al*, 2005). In the present study, therefore, we opted for the 2-week protocol. The reason we administered docetaxel and CDDP on day 8 and not on day 1, as in previously reported S-1/CDDP combination studies (Ajani *et al*, 2005b), was that when we, in the pilot study, administered both drugs on day 1, we encountered severe neutropenia on days 10–14. We used an S-1 dosage of 40 mg m^−2^ twice daily in this study according to the previous studies (Koizumi *et al*, 2000; Yoshida *et al*, 2006).

Although this is a phase I study recruiting only 17 patients, the RR was quite high (88%) compared with those of phase II and phase III studies previously reported. Taking such a high RR into account, one may assume that the results in terms of TTP (199 days) and MST (367 days) are not impressive. However, considering that this study included 11 patients with distant metastases to liver, peritoneum and bone, which are reportedly poor prognostic factors (Chau *et al*, 2004; Kim *et al*, 2007), the present results of TTP and MST are, we believe, reasonably acceptable. An important finding in this study was that there were no cases of PD, unlike in other previous studies. Furthermore, the most noteworthy result of our study was that four of the 17 patients (23.5%) underwent downstaging, showing a rapid reduction of the sizes of tumours. As a mechanism of the rapid tumour regression in these cases, we have postulated that our regimen has an antiangiogenic effect, since it has been reported that docetaxel inhibits tumour angiogenesis by interfering with growth, migration and tubule formation of endothelial cells (Hotchkiss *et al*, 2002), and that the combination of S-1 with CDDP has shown a high RR in VEGF-positive gastric cancer (Hironaka *et al*, 2002). This hypothesis was supported by immunohistochemical examination for VEGF disclosing a significantly stronger staining in downstaged patients than in non-downstaged patients.

Generally, neoadjuvant chemotherapy for stage II/III gastric cancer is still unacceptable because some cases undergo PD, thereby leading to an inoperable status during the chemotherapy before gastrectomy. However, the fact that we observed no PD patients along with a certain appreciable rate of downstaging in this study suggests the applicability of our regimen to neoadjuvant chemotherapy. Therefore, a clinical trial of neoadjuvant chemotherapy and a large-scale phase II study of our regimen are currently underway.

In conclusion, this phase I study revealed the feasibility of the triple combination of docetaxel, S-1 and CDDP. The DLT was neutropenia. Our regimen indicated a very high response (88%, 15/17) in patients with unresectable metastatic gastric cancer. More importantly, four of the 17 patients (23.5%) achieved downstaging and underwent subsequent curative gastrectomy. The active mechanism of our regimen in these four patients is suggested to involve antiangiogenic activity.

## Figures and Tables

**Table 1:** Patient characteristics

**Characteristics**	**Number of patients**
*Total*	17
Male	12
Female	5
	
*Age (years)*	
Median	61
Range	54–75
	
*Performance status*	
0	5
1	11
2	1
	
*Histology*	
Intestinal type	6
Diffuse type	11
	
*Metastatic sites*	
LNs	17
Distant LN^a^	14
Liver	4
Peritoneum	4
Bone	3
Lung	1

LN=lymph node.

a Seven patients with para-aortic lymph node, five patients with Virchow lymph node and two patients with mediastinal lymph node metastases.

**Table 2:** Toxicities during the first course

			**Grade of side effects**		
**Toxicity**	**Dose level**	**Number of patients**	**2**	**3**	**4**
*Haematological toxicity*					
Leukocytopenia	1	9	2	3	0
	2	5	2	1	2
	3	3	1	2	1
Neutropenia	1	9	3	3	0
	2	5	1	2	2
	3	3	0	2	2
Anaemia	1	9	1	0	0
	2	5	0	0	0
	3	3	1	0	0
Thrombocytopenia	1	9	0	0	0
	2	5	0	0	0
	3	3	0	0	0
					
*Non-haematological toxicity*					
Nausea	1	9	3	0	0
	2	5	1	1	0
	3	3	0	2	0
Vomiting	1	9	0	0	0
	2	5	1	0	0
	3	3	0	0	0
Diarrhoea	1	9	0	0	0
	2	5	0	0	0
	3	3	1	0	0
Infection	1	9	0	0	0
	2	5	0	0	0
	3	3	0	0	0
GOT/GPT elevation	1	9	0	0	0
	2	5	0	0	0
	3	3	0	0	0
Creatinine elevation	1	9	0	0	0
	2	5	0	0	0
	3	3	1	0	0

**Table 3:** Toxicities during all courses

			**Grade of side effects**		
**Toxicity**	**Dose level**	**Number of patients**	**2**	**3**	**4**
*Haematological toxicity*					
Leukocytopenia	1	9	4	4	1
	2	5	1	1	2
	3	3	0	2	2
Neutropenia	1	9	3	4	2
	2	5	0	2	3
	3	3	0	2	2
Anaemia	1	9	2	0	0
	2	5	0	0	0
	3	3	1	0	0
Thrombocytopenia	1	9	1	0	
	2	5	0	0	0
	3	3	0	0	0
					
*Non-haematological toxicity*					
Nausea	1	9	4	0	0
	2	5	1	1	0
	3	3	1	2	1
Vomiting	1	9	1	0	0
	2	5	1	0	0
	3	3	1	0	0
Diarrhoea	1	9	2	0	0
	2	5	2	0	0
	3	3	1	0	0
Infection	1	9	1	0	0
	2	5	1	1	0
	3	3	1	0	0
GOT/GPT elevation	1	9	1	0	0
	2	5	1	0	0
	3	3	0	0	0
Creatinine elevation	1	9	1	0	0
	2	5	0	0	0
	3	3	1	0	0

**Table 4:** Objective response

		**Response**						**TTP (day)**		
**Dose level**	**Number of patients**	**CR**	**PR**	**SD**	**PD**	**Response rate (%)**	**Number of downstaged patients (%)**	**Median**	**Range**	**3-year survival (%)**
1	9	0	8	1	0	88.9	2 (22.2)	226	104–1130	2 (22.2)
2	5	1	3	1	0	80.0	1 (20.0)	192	152–1097	1 (20.0)
3	3	0	3	0	0	100.0	1 (33.3)	231	120–833	1 (33.3)
Overall	17	1	14	2	0	88.2	4 (23.5)	199	104–1130	4 (23.5)

CR=complete response; PD=progressive disease; PR=partial response; SD=stable disease; TTP=time to progression.
